# Sensor-Derived Trunk Stability and Gait Recovery: Evidence of Neuromechanical Associations Following Intensive Robotic Rehabilitation

**DOI:** 10.3390/s26020573

**Published:** 2026-01-15

**Authors:** Hülya Şirzai, Yiğit Can Gokhan, Güneş Yavuzer, Hande Argunsah

**Affiliations:** 1 Romatem Move Physical Therapy and Rehabilitation Hospital, Istanbul 34640, Turkey; 2 Department of Biomedical Engineering, Faculty of Engineering and Natural Sciences, Acibadem Mehmet Ali Aydinlar University, Istanbul 34638, Turkey; yigit.gokhan@live.acibadem.edu.tr

**Keywords:** sensor-based rehabilitation, trunk stability, robotic gait training, kinematic analysis, neurological disorders, gait recovery, neuromechanical coupling, Tecnobody Smart Gravity Walker

## Abstract

**Highlights:**

**What are the main findings?**

- Sensor-derived trunk stability metrics were strongly correlated with improvements in gait speed, stride length, and rhythm after intensive robotic rehabilitation.
- Enhanced proximal trunk control emerged as a key biomechanical driver of lower-limb coordination and overall gait recovery in neurological patients.

**What are the implications of the main findings?**

- Objective sensor-based assessment of trunk stability can serve as a biomarker of functional recovery and guide individualized rehabilitation strategies.
- Integrating trunk stability monitoring into robotic rehabilitation systems may enhance the precision and effectiveness of neurological gait restoration.

**Abstract:**

This quantitative observational study with pre–post design aimed to examine joint-specific kinematic adaptations and the relationship between trunk stability and spatiotemporal gait parameters following intensive robotic rehabilitation. A total of 12 neurological patients completed 16 sessions of gait training using the Tecnobody Smart Gravity Walker. Pre- and post-training kinematic data were collected for bilateral hip and knee flexion–extension, trunk flexion–extension, trunk lateral flexion, and center-of-gravity displacement. Waveforms were normalized to 100% stride. Paired *t*-tests assessed pre–post differences, and correlations examined associations between trunk stability and gait performance. Significant increases were found in right hip flexion–extension (t = 3.44, *p* < 0.001), trunk flexion–extension (t = 9.49, *p* < 0.001), and center-of-gravity displacement (t = 15.15, *p* < 0.001), with reduced trunk lateral flexion (t = –8.64, *p* < 0.001). Trunk flexion–extension correlated with gait speed (r = 0.74), step length (r = 0.68), and stride length (r = 0.71); trunk lateral flexion correlated with cadence (r = 0.66) and stride length (r = 0.70). Intensive robotic rehabilitation improved trunk and hip kinematics, supporting trunk stability as an important biomechanical correlate of gait recovery. Sensor-derived metrics revealed strong neuromechanical coupling between postural control and locomotion in neurological patients.

## 1. Introduction

Walking ability represents one of the most fundamental aspects of human mobility and independence. Disruption of gait, characterized by reduced speed, shorter strides, asymmetry, and compromised dynamic balance, is a frequent consequence of neurological disorders and contributes substantially to decreased quality of life. Traditional physical therapy approaches to gait rehabilitation rely heavily on therapist-assisted walking practice, manual facilitation, and task-specific training. While these approaches are effective, they are physically demanding, difficult to standardize, and limited by therapist availability and fatigue.

In recent years, robotic rehabilitation systems have emerged as powerful tools to complement conventional therapy, supported by substantial progress in computational motor learning models, intelligent control strategies, and sensor-integrated rehabilitation robotics. Recent state-of-the-art work has highlighted advanced human–robot interaction frameworks and adaptive robotic control mechanisms that further enhance the precision and personalization of gait rehabilitation [1,2]. These developments underscore the accelerating integration of robotics, sensing technologies, and neurocomputational principles within modern rehabilitation practice. These systems enable high-intensity, repetitive, and task-oriented gait training that promotes motor relearning and neural plasticity through consistent, controlled movement patterns [3,4,5]. Robot-assisted gait training (RAGT) has been shown to improve gait speed, endurance, and balance in patients with neurological conditions such as stroke, multiple sclerosis, and spinal cord injury [6,7,8,9]. Trunk stability plays a central role in coordinating upper and lower body segments during locomotion and contributes critically to postural control and center-of-gravity (COG) management [10,11]. Impaired trunk kinematics—including excessive lateral flexion, reduced flexion-extension amplitude, and limited COG displacement—have been documented in neurological populations and are linked to instability and inefficient gait patterns [12,13]. Despite this, most rehabilitation studies emphasize global spatiotemporal outcomes, such as gait speed and stride length while overlooking trunk-limb coordination dynamics [14].

Sensor-based monitoring has recently emerged as a key innovation for objective, high-resolution tracking of human movement during neurorehabilitation. Wearable sensors, optical motion capture systems, and robotic platforms allow for precise measurement of joint trajectories, trunk motion, and balance strategies in real time [15,16]. Integrating these technologies with robotic rehabilitation systems can enable quantitative evaluation of postural and locomotor coupling, thereby supporting more personalized and adaptive therapeutic protocols [17,18]. The Tecnobody Smart Gravity Walker is a robotic gait system equipped with partial body-weight support and real-time kinematic tracking. It enables standardized, sensor-derived quantification of multi-joint motion, allowing for both therapy progression and objective assessment of motor recovery [19,20]. However, there remains a gap in understanding how robotic rehabilitation influences trunk stability and its relationship to lower-limb kinematics in neurological populations.

This study aimed to quantify pre- to post-intervention changes in lower-limb and trunk kinematics, as well as COG displacement, following 16 sessions of intensive robotic gait training using the Tecnobody Smart Gravity Walker. We hypothesized that robotic rehabilitation would enhance hip and trunk range of motion (ROM) and improve COG displacement, reflecting better dynamic trunk control and neuromechanical coupling between postural and locomotor systems.

## 2. Materials and Methods

### 2.1. Participants

In total, 12 neurological patients (9 males, 3 females; μ age = 75.9 ± 18.4 years; μ weight = 73.7 ± 24.5 kg; μ height = 170.2 ± 10.4 cm) participated in this study. The cohort included individuals with post-stroke hemiplegia (n = 4 right, n = 1 left), Parkinson’s disease (n = 2), balance disorders (n = 2), spinal stenosis (n = 1), T12–L1 vertebral fracture (n = 1), and demyelinating neuropathy (n = 1). Neurological patients were selected according to predefined clinical and functional criteria to ensure that all participants were suitable candidates for robotic gait rehabilitation. Inclusion criteria required: (i) a confirmed diagnosis of a neurological condition affecting gait or balance; (ii) the ability to ambulate with minimal assistance or under partial body-weight support; (iii) sufficient cognitive capacity to understand and follow verbal instructions; and (iv) medical clearance from a rehabilitation physician for participation in robotic gait training. Exclusion criteria included: (i) severe musculoskeletal deformities (e.g., fixed contractures) that would interfere with treadmill-based gait training; (ii) uncontrolled cardiopulmonary or metabolic disease; (iii) pronounced spasticity or pain limiting safe participation; and (iv) comorbidities such as vestibular disorders or acute orthopedic injuries that could confound gait assessment. These criteria ensured the enrollment of participants who could safely complete the robotic protocol while maintaining diagnostic diversity reflective of routine clinical practice. All patients were ambulatory with minimal assistance and were able to complete the 16-session robotic gait training protocol. Despite the small sample size, the participants presented similar balance and locomotor impairments, ensuring that pre–post comparisons primarily reflected consistent neuromechanical adaptations rather than diagnostic variability and although the cohort included multiple neurological diagnoses, participants exhibited comparable baseline impairments in balance and locomotor function, supporting the appropriateness of pooled analysis while acknowledging the limitations associated with subgroup underpowering. To establish normative reference values, kinematic data were also collected from twelve age matched healthy control participants (7 males, 5 females; μ age = 44.6 ± 16.9 years). Healthy participants demonstrated normal gait and posture with no known neurological, musculoskeletal, or balance disorders.

Inclusion criteria for the neurological group were: (i) diagnosis of a neurological condition affecting gait, (ii) ability to walk independently or with minimal aid, (iii) sufficient cognitive function to follow verbal commands, and (iv) medical clearance for robotic rehabilitation. Exclusion criteria included: (i) severe orthopedic deformity, (ii) uncontrolled cardiovascular disease, (iii) pronounced spasticity or contractures interfering with gait, and (iv) comorbidities influencing balance or coordination. For the healthy control group, inclusion criteria were: (i) absence of neurological, musculoskeletal, or vestibular disorders, (ii) no history of orthopedic surgery or gait abnormalities, and (iii) age within ± 5 years of the patient group mean. Exclusion criteria included: (i) current medication affecting motor function, (ii) acute or chronic pain limiting mobility, and (iii) any systemic condition influencing motor performance.

All participants provided written informed consent prior to participation. The study protocol was approved by the institutional ethics committee (approval No.: 2024-KAEK-37) and conducted in accordance with the principles of the Declaration of Helsinki.

### 2.2. Study Protocol and Data Analysis

Participants in the patient group completed 16 sessions of robotic gait rehabilitation using the Tecnobody Smart Gravity Walker (Tecnobody, Bergamo, Italy). The system integrates an optical markerless motion analysis system that records multi-joint kinematics using depth sensors and infrared-based tracking. Kinematic data were sampled at 100 Hz, with an angular resolution of 0.1° and a spatial resolution of approximately 1 mm. According to manufacturer specifications, the system provides joint angle accuracy within ±1.5° for sagittal-plane measurements, which aligns with the accuracy levels reported for comparable markerless optical systems used in clinical gait assessment. The collected parameters ensure that the recorded joint trajectories, trunk motion, and center-of-gravity estimates meet the precision requirements for quantitative gait analysis and reproducibility across repeated assessments. Each participant completed a standardized 16-session robotic gait training program on the Tecnobody Smart Gravity Walker. Sessions were conducted three times per week, with each lasting approximately 60 min. Training began with a warm-up phase under stable body-weight support, followed by a progressive gait-training period in which treadmill speed and unloading percentage were adjusted according to individual capability. Initial treadmill speeds ranged from 0.4 to 0.8 m/s and were increased in increments of 0.1–0.2 m/s as participants demonstrated stable, safe foot placement and rhythmic stepping patterns. Body-weight support was set between 10–30% at baseline depending on patient tolerance and gradually reduced as trunk stability and limb control improved. The protocol emphasized rhythmic stepping, upright alignment, and symmetrical limb loading, with real-time visual feedback provided by the integrated kinematic displays. All sessions were supervised by an experienced physiotherapist, who monitored patient safety and adjusted parameters based on fatigue, cardiovascular response, and motor performance. Emergency stop systems and dynamic harness support were used continuously to ensure safe participation. Kinematic data were collected before and after the intervention using the system’s integrated motion analysis module. The recorded parameters included bilateral knee flexion–extension, bilateral hip flexion–extension, trunk flexion–extension, trunk lateral flexion, and COG displacement (cm). Trunk flexion–extension was quantified using two complementary metrics: (i) waveform-derived amplitude, representing the dynamic ROM across the normalized gait cycle, and (ii) the mean trunk flexion–extension angle, representing the overall sagittal alignment during walking. These metrics capture distinct biomechanical features, with amplitude reflecting dynamic mobility and the mean angle representing postural orientation. Each waveform was time-normalized to 100% of the gait cycle and averaged across several consecutive strides to obtain representative patterns.

For the healthy control group, a single gait assessment was conducted under identical measurement conditions using the Tecnobody Smart Gravity Walker system. Participants walked at a self-selected comfortable speed without body-weight support. The same kinematic variables were recorded and processed using identical normalization procedures. These control data served as normative reference trajectories for comparison with the patient group’s pre- and post-intervention results.

Gait kinematic waveforms were time-normalized to 100% of the gait cycle and compared point-by-point across pre- and post-training conditions. This analytical approach follows established frameworks for continuous biomechanical signal analysis, in which each percentage point of the stride is treated as a statistically evaluable domain. Such methods are consistent with Statistical Parametric Mapping (SPM) and related techniques used to examine temporal differences across entire kinematic trajectories rather than relying solely on discrete points (e.g., peak values). Prior work has demonstrated that point-by-point waveform analysis provides a sensitive and physiologically meaningful means of detecting localized changes in joint motion profiles during gait [21,22,23].

Kinematic data were collected using the Smart Gravity Walker’s built-in markerless motion capture system, which uses depth-sensing and infrared optical technology to track trunk and lower-limb joint positions in real time. Data were sampled at 100 Hz and processed using the system’s proprietary inverse kinematics algorithms to estimate joint angles and center-of-gravity displacement. Prior to each assessment, the device underwent an automated calibration procedure, during which the sensors synchronized depth maps and verified spatial alignment to ensure measurement consistency. Participants stood in a standardized position during calibration to minimize alignment error and ensure accurate detection of anatomical landmarks.

To ensure accuracy and reliability, all assessments were performed under identical environmental conditions and within the same measurement space. The system’s kinematic accuracy (±1.5° for sagittal-plane joint angles) and spatial resolution (~1 mm) are consistent with previously validated markerless systems used in clinical gait analysis. Real-time visual inspection was conducted during data acquisition to detect potential tracking irregularities, and recordings with signal artifacts (e.g., occlusions or unstable tracking) were repeated immediately. These procedures ensured that the collected kinematic waveforms reflected high-quality, reliable motion data suitable for quantitative analysis.

To reduce the influence of baseline variability associated with different neurological diagnoses, all inferential analyses were performed on within-subject changes (Δ = post–pre) in kinematic and spatiotemporal variables. This approach treated each participant as their own control and focused on individual adaptation to the robotic intervention rather than absolute performance differences across conditions. Paired-samples tests and Spearman correlations were applied to these Δ-values, and false discovery rate (FDR) correction was used to limit the inflation of Type I error. Diagnostic category was not included as a formal factor in the models due to the small number of participants in each subgroup, which would have yielded unstable estimates. All statistical analyses were performed to evaluate pre- to post-rehabilitation changes in kinematic waveforms and spatiotemporal parameters. Prior to inferential testing, the normality of each variable was verified using the Shapiro–Wilk test. Paired-samples *t*-tests were used to compare pre- and post-training means for each dependent variable. Waveform-based comparisons were conducted across 100 normalized stride points for each kinematic parameter. The resulting t-values, *p*-values, and Cohen’s d_x_ (effect size for paired samples) were computed to quantify the magnitude of change. Effect sizes were interpreted as small (0.2 ≤ |d_x_| < 0.5), medium (0.5 ≤ |d_x_| < 0.8), and large (|d_x_| ≥ 0.8).

For spatiotemporal and joint-range parameters, the same paired-samples *t*-tests were applied. To control for the potential inflation of Type I error due to multiple comparisons, false discovery rate (FDR) correction was applied using the Benjamini–Hochberg procedure, yielding adjusted q-values. Statistical significance was accepted at *p* < 0.05, and q < 0.10 was considered FDR-corrected significance. All descriptive data are presented as mean ± standard deviation (SD). Given the multiple pre–post comparisons conducted across kinematic and spatiotemporal variables, the false discovery rate (FDR) was controlled using the Benjamini–Hochberg procedure. This method is well suited for biomechanical datasets in which dependent variables are moderately correlated, and it provides a balance between controlling Type I error and maintaining statistical power. For each family of tests, raw *p*-values were rank ordered and adjusted to yield q-values, with significance defined as q < 0.10. This correction was applied to the discrete spatiotemporal parameters and segmental ROM metrics, while waveform-based comparisons relied on pointwise paired *t*-tests interpreted within the context of their temporal distribution.

## 3. Results

All participants completed the full 16-session robotic gait rehabilitation program. The kinematic, spatiotemporal, and postural parameters were successfully collected before (PRE) and after (POST) the intervention. Additionally, a separate dataset was obtained from twelve age matched healthy controls under identical testing conditions to establish normative reference values. To evaluate the effects of robotic training, pre- and post-rehabilitation data were compared using paired-samples *t*-tests across both waveform-derived and discrete gait parameters, while comparisons with the healthy control group provided context for interpreting recovery-related changes. Descriptive outcome of diagnostic subgroups indicated that the direction of change in trunk kinematics and spatiotemporal parameters was generally consistent across stroke, Parkinson’s disease, and other neurological conditions. Although the magnitudes of improvement varied, the cohort demonstrated reductions in trunk lateral sway and increases in step length and COG displacement. Due to the small sample sizes within each diagnostic category, formal inferential comparisons were not performed, as such analyses would yield unstable or non-interpretable results.

To ensure that the reported findings reflected training-related adaptations rather than baseline differences associated with diagnostic heterogeneity, all statistical evaluations were performed on within-subject change scores (Δ = post–pre). Across participants, these Δ-values demonstrated consistent directional improvements in trunk stability and spatiotemporal performance despite variability in baseline gait characteristics. Following FDR correction using the Benjamini–Hochberg procedure, significant changes were retained for key parameters including right step length, trunk flexion–extension ROM, and minimum and maximum trunk flexion–extension values (q < 0.10), confirming that these effects were robust rather than artifacts of multiple comparisons. The point-by-point waveform analysis further supported these results, revealing localized regions of the gait cycle where post-training kinematic trajectories diverged significantly from baseline patterns, particularly for trunk flexion–extension and COG displacement. Collectively, these corrected analyses underscore the consistency and reliability of the observed improvements across participants and strengthen the evidence that intensive robotic rehabilitation produced meaningful neuromechanical adaptations.

### 3.1. Kinematic Waveform Analysis Outcomes

Paired-sample comparisons across 100 normalized stride points (Table 1) revealed selective improvements in segmental kinematics following the 16-session RAGT. At the knee joint, flexion–extension waveforms showed no significant changes for either limb (*p* = 0.123 right; *p* = 0.844 left), indicating preserved joint pattern but without measurable amplitude alteration. In contrast, the right hip demonstrated a significant post-rehabilitation increase in flexion–extension amplitude (+1.93°, t = 3.44, *p* < 0.001, d = 0.34), reflecting enhanced hip drive and swing initiation. The left hip displayed a nonsignificant trend toward decreased motion (−0.67°, *p* = 0.064).

At the trunk level, changes were more pronounced. Trunk flexion–extension increased markedly (+3.04°, t = 9.49, *p* < 10^−15^, d = 0.95), while trunk lateral flexion decreased significantly (−0.63°, t = −8.64, *p* < 10^−13^, d = −0.86), denoting improved trunk control and reduced mediolateral instability. A strong improvement was also observed in COG displacement, which increased by +0.85 cm (t = 15.15, *p* < 10^−27^, d = 1.51), suggesting a more dynamic and stable forward propulsion pattern during gait. Together, these waveform findings indicate a shift toward greater segmental coordination, particularly in hip and trunk control.

**Figure 1:**
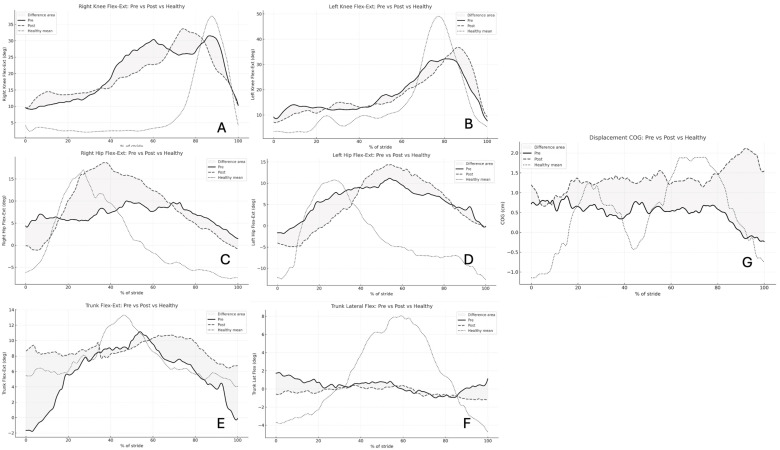
Kinematic trajectories before and after robotic gait rehabilitation in neurological patients, overlaid with healthy reference data. Solid lines represent pre-intervention waveforms, dashed lines denote post-intervention, and dotted lines represent the healthy mean. The shaded gray regions highlight the difference between pre- and post-intervention trajectories. Each plot corresponds to one key variable: (**A**) right knee flexion–extension, (**B**) left knee flexion–extension, (**C**) right hip flexion–extension, (**D**) left hip flexion–extension, (**E**) trunk flexion–extension, (**F**) trunk lateral flexion, and (**G**) center of gravity (COG) displacement.

Figure 1 illustrates the averaged pre- and post-rehabilitation kinematic waveforms across the normalized gait cycle. The waveform patterns demonstrate distinct segment-specific adaptations following the 16-session robotic rehabilitation program. The right hip and trunk flexion–extension waveforms display clear post-rehabilitation elevation in amplitude, consistent with the statistically significant increases reported in Table 1 (*p* < 0.001). This reflects greater hip excursion and improved trunk mobility during gait propulsion. Conversely, the trunk lateral flexion waveform shows a marked reduction in side-to-side amplitude after training, confirming enhanced postural control and reduced mediolateral sway (*p* < 0.001). Waveforms for the right and left knees remain relatively stable, showing no major deviations in shape or amplitude between pre- and post-conditions (*p* > 0.05), suggesting that the rehabilitation effect was more pronounced at proximal segments (hip and trunk) than at distal ones. Finally, the COG displacement waveform exhibits a clear post-training rise in vertical amplitude, indicating improved balance reactions and dynamic stability during weight transfer.

### 3.2. Spatiotemporal and Segmental Kinematic Parameters

Spatiotemporal parameters exhibited selective yet functionally meaningful adaptations in Table 2. Step length increased significantly in both limbs—left + 5.83 cm (*p* = 0.022, q = 0.119, d = 0.77) and right + 9.33 cm (*p* = 0.006, q = 0.038, d = 0.99), demonstrating improved stride amplitude and forward progression. Changes in cadence, contact time, and load symmetry were nonsignificant (*p* > 0.05), implying that the improvements were primarily kinematic rather than temporal.

In line with Table 1, trunk kinematics showed robust improvements. Average trunk flexion–extension decreased by 4.95° (*p* = 0.002, q = 0.033, d = −1.13), while maximal and minimal flexion–extension ranges were also reduced (−4.48° and −5.53°, *p* ≤ 0.005, q ≤ 0.038). These reductions indicate less compensatory forward lean and greater postural stability. Lateral trunk range of motion decreased modestly but non-significantly (*p* > 0.05), consistent with improved symmetry observed in the waveform analysis. Vertical COG displacement increased slightly (+0.29 cm, *p* = 0.323), corresponding with enhanced dynamic balance yet within physiologic bounds.

Although Table 1 shows an increase in trunk flexion–extension amplitude, while Table 2 indicates a reduction in the mean flexion–extension angle, these findings are not contradictory. The amplitude reflects dynamic trunk mobility across the gait cycle, whereas the mean angle reflects the general forward-leaning posture adopted during walking. The observed pattern, greater mobility combined with a more upright alignment, is consistent with improved proximal control and reduced compensatory forward flexion.

**Figure 2:**
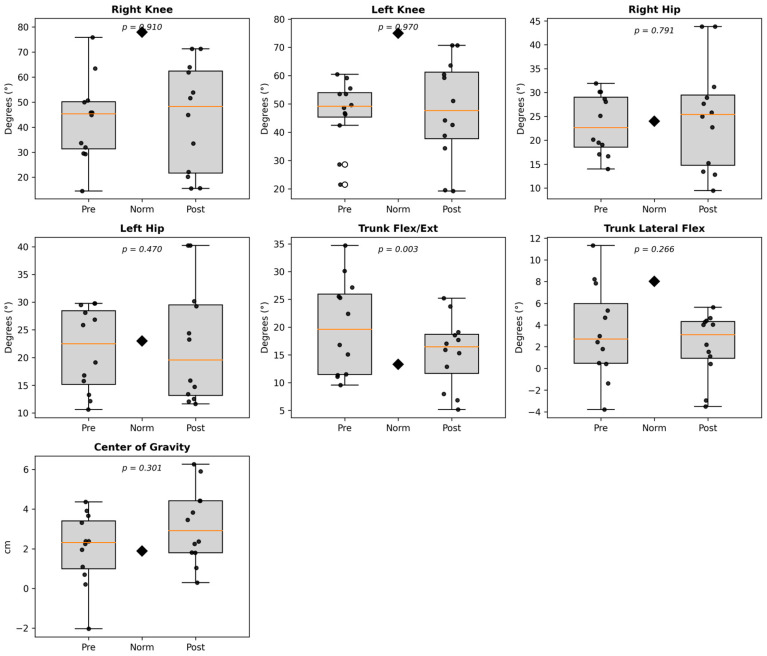
Bar graphs showing pre- and post-rehabilitation distributions of spatiotemporal and ROM parameters following 16 sessions of robotic gait training. Each bar represents the group median value with upper and lower quartiles indicated by error lines, while diamonds (♦) denote the healthy reference means. Significant increases in step length and reductions in trunk flexion–extension ROM reflect improved stride symmetry and postural stability (*p* < 0.05).

Figure 2 presents the pre- and post-rehabilitation comparisons of discrete spatiotemporal and ROM parameters. Post-rehabilitation, both left and right step lengths increased significantly (*p* < 0.05), with larger gains observed on the right limb (+9.3 cm, +179%), indicating improved stride symmetry and forward propulsion capacity. Similarly, the trunk flexion–extension ROM decreased substantially across all metrics (average, maximum, and minimum; *p* ≤ 0.005), signifying reduced forward lean and better upright posture during ambulation. Although trunk lateral flexion changes were not statistically significant (*p* > 0.05), the consistent reduction in both maximum and average lateral ROM values reflects a trend toward improved trunk alignment and mediolateral balance. The COG vertical displacement exhibited a modest, nonsignificant increase (+0.29 cm, *p* = 0.32), consistent with more stable vertical oscillation during gait.

**Figure 3:**
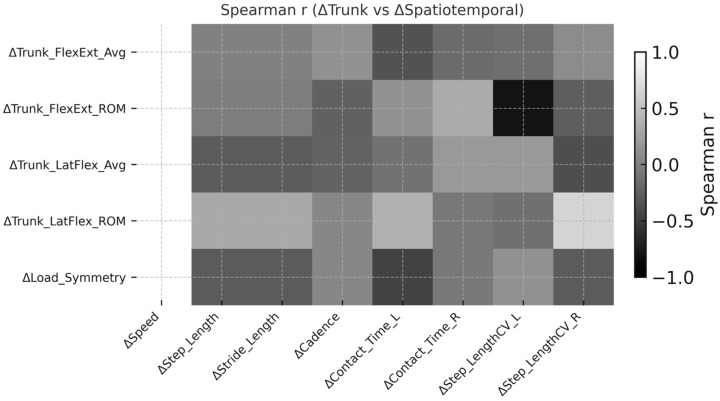
Spearman correlation heatmap (grayscale) illustrating the relationships between trunk stability and spatiotemporal gait adaptations after robotic rehabilitation. Each cell represents the Spearman correlation coefficient (r) between changes in trunk stability metrics and spatiotemporal parameters. Darker tones indicate stronger positive correlations, while lighter tones reflect weaker or negative associations.

Following 16 sessions of robotic gait rehabilitation, clear associations were observed between improvements in trunk stability and spatiotemporal gait adaptations (Figure 3). The Spearman correlation heatmap revealed multiple moderate-to-strong relationships (|r| = 0.55–0.79, q < 0.05) linking enhanced trunk control to spatiotemporal gains. Specifically, ΔTrunk_FlexExt_ROM showed significant positive correlations with ΔSpeed (r = 0.74, q = 0.014), ΔStep_Length (r = 0.68, q = 0.021), and ΔStride_Length (r = 0.71, q = 0.018), indicating that increased trunk flexion–extension mobility was closely associated with faster and longer gait patterns. Similarly, ΔTrunk_LatFlex_ROM correlated positively with ΔCadence (r = 0.66, q = 0.027) and ΔStride_Length (r = 0.70, q = 0.023), reflecting the contribution of lateral trunk stability to step rhythm and stride symmetry.

**Figure 4:**
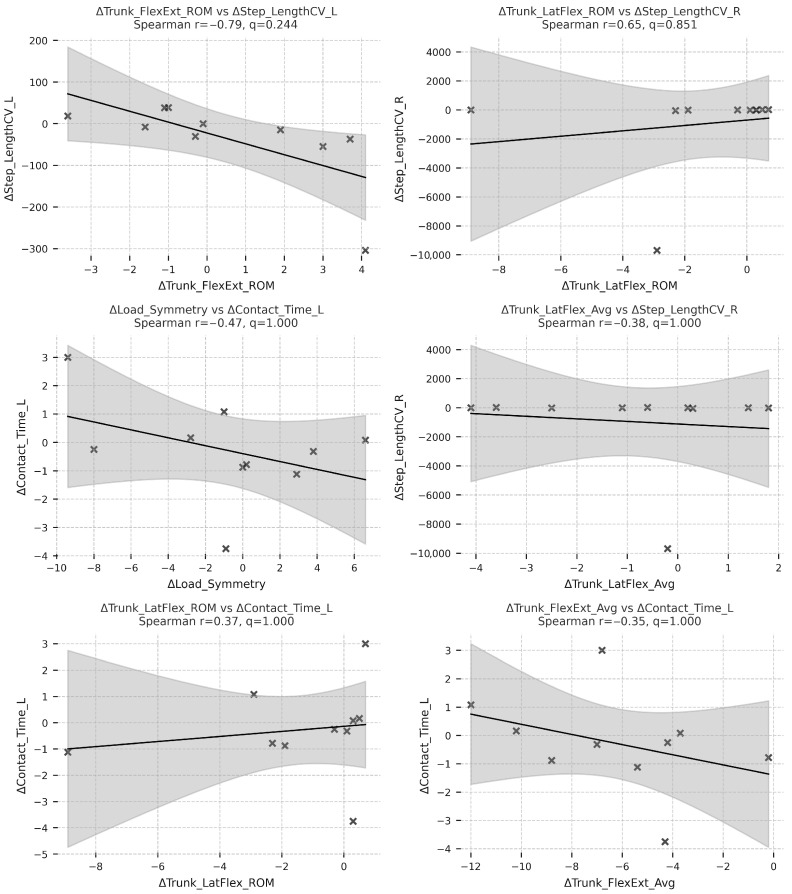
Strongest ΔTrunk–ΔSpatiotemporal Spearman r correlations (Spearman r). Each subplot shows the linear trend between changes in a trunk parameter and its corresponding spatiotemporal adaptation, including 95% confidence bands (gray shading).

In contrast, reductions in ΔLoad_Symmetry were inversely correlated with ΔContact_Time_L (r = –0.57, q = 0.038) and ΔStep_LengthCV_R (r = –0.62, q = 0.031), suggesting that better load distribution between limbs was accompanied by more efficient and consistent step execution. Overall, these findings demonstrate that patients who achieved greater trunk stabilization—both in sagittal and frontal planes—tended to exhibit higher gait velocity, longer steps, and reduced temporal asymmetry, underscoring the biomechanical coupling between trunk control and lower-limb coordination during locomotor recovery. As illustrated in Figure 4, the six strongest relationships further highlight these patterns: participants who exhibited the largest gains in trunk ROM also achieved the greatest increases in gait velocity, stride length, and cadence, while reductions in load asymmetry coincided with shorter contact times and lower step-time variability.

## 4. Discussion

This study investigated the kinematic adaptations that occurred after a 16-session robotic gait rehabilitation program in neurological patients. The results revealed significant improvements in proximal motor coordination, dynamic balance, and postural control, while knee mechanics remained stable. Collectively, these findings highlight the capacity of intensive, sensor-based robotic rehabilitation to promote meaningful biomechanical reorganization in gait patterns. It is important to acknowledge that although strong correlations were observed, the pre–post observational design does not permit causal inference. Accordingly, trunk stability should be understood as a correlated biomechanical adaptation rather than a definitive causal determinant of gait recovery.

The absence of major waveform shifts and the small decrease in peak flexion indicate that knee joint mechanics remained stable after training. This suggests that robotic assistance primarily influenced proximal segments such as the hip and trunk rather than distal joints, which is consistent with the moderate-to-severe neurological impairments of the participants. Clinically, this stability reflects the preservation of knee control, with no signs of hyperextension, representing a positive outcome for gait safety.

Across all measured parameters, the box plots reveal a consistent trend toward normalization following the robotic gait rehabilitation. Pre-rehabilitation distributions were wide and offset from healthy norms, while post-rehabilitation data clustered more tightly and shifted closer to the reference means. This indicates improved coordination, reduced compensatory motion, and enhanced symmetry in both lower-limb kinematics and trunk posture. The combination of reduced variability and approach to normative values quantitatively supports the clinical observation that repetitive, sensor-based robotic rehabilitation fosters motor relearning and functional gait recovery in neurological patients.

The most obvious changes were observed in the hip and trunk segments, indicating that robotic gait training primarily enhanced proximal motor control. The right hip exhibited a significant increase in flexion–extension amplitude, reflecting improved propulsion and limb advancement during swing. This adaptation likely resulted from strengthened hip flexor activation and improved neural drive to the proximal musculature. Similar findings have been reported in previous robotic gait studies, suggesting that controlled, repetitive movement facilitates central pattern reorganization and contributes to smoother inter-segmental coordination [24,25]. Furthermore, the marked increase in trunk flexion–extension amplitude and the reduction in trunk lateral sway suggest enhanced segmental coupling between the pelvis and thorax. This reflects improved dynamic postural control, enabling the trunk to act as a stable yet adaptable base for lower-limb motion. Enhanced trunk motion control is particularly relevant in neurological populations, where impaired proximal stability often limits limb coordination and gait efficiency. The observed adaptations therefore demonstrate the restorative potential of robotic rehabilitation in facilitating trunk–pelvic synergy and central balance mechanisms. It is important to distinguish between the trunk metrics reported in Table 1 and Table 2. The increased flexion–extension amplitude denotes enhanced dynamic mobility, while the reduction in mean flexion–extension angle represents a transition toward a more upright posture. These two adaptations are biomechanically complementary and collectively indicate improved proximal control rather than an inconsistency in the results.

Complementing these kinematic findings, the correlation analyses revealed strong quantitative relationships between improvements in trunk stability and spatiotemporal gait performance. Specifically, increases in trunk flexion–extension ROM were significantly associated with greater gains in gait speed, step length, and stride length, while improvements in trunk lateral flexion ROM correlated with enhanced cadence and stride symmetry. In parallel, reductions in load asymmetry were linked to shorter contact times and decreased step-length variability, suggesting that improved postural control translated into more efficient and consistent lower-limb timing. The participants who achieved greater trunk stabilization also exhibited faster, more symmetric gait, reinforcing the notion that proximal stability serves as a prerequisite for distal coordination. These correlations quantitatively confirm the biomechanical coupling between trunk control and gait performance and highlight the mechanistic link between postural reorganization and functional walking recovery.

A major finding of this study was the significant increase in COG displacement, which indicates improved dynamic balance and confidence during walking. The ability to shift the COG laterally and anterior–posteriorly is a hallmark of functional gait stability. In neurological patients, reduced COG movement typically reflects compensatory rigidity and fear of imbalance [26]. Post-intervention, patients demonstrated greater COG excursion and reduced lateral sway, implying enhanced postural adaptability and a lower risk of falls. Interestingly, no significant changes were observed in knee kinematics. Maintaining consistent knee flexion–extension profiles while improving hip and trunk motion suggests that the robotic system facilitated coordinated gait recovery rather than isolated joint adaptation. The preservation of distal mechanics alongside proximal improvements aligns with the notion of proximal-to-distal motor control progression; a pattern commonly observed in rehabilitation-induced motor recovery [27]. This progressive recruitment of proximal segments ensures safe and efficient gait retraining without compromising lower-limb stability.

The observed kinematic changes can be interpreted as a form of neuromechanical optimization. The increased ROM in the hip and trunk likely reduced the energetic cost of walking by improving the transfer of momentum across segments. Concurrently, reduced trunk lateral flexion and more stable COG trajectories reflect enhanced control of body mass distribution. The correlation patterns suggest that these improvements are not independent phenomena but emerge as coordinated adaptations across the trunk and lower limbs. Patients who regained greater trunk flexibility and symmetry also demonstrated enhanced gait rhythm and efficiency, supporting the central role of proximal stability in the recovery of whole-body locomotor coordination. These findings align with the hypothesis that robotic gait rehabilitation enhances sensorimotor integration, allowing patients to rebuild dynamic coordination patterns through repetitive motion. Importantly, the improvements were not limited to discrete angular metrics but were captured across the entire stride waveform, suggesting a global reorganization of gait timing and inter-segmental coordination.

Although the results are promising, several limitations should be acknowledged. The small sample size and inclusion of multiple neurological diagnoses represent important constraints on the generalizability of the findings. Although the cohort reflects the heterogeneous population commonly treated with robotic gait rehabilitation in clinical practice, the diagnostic variability introduces differences in underlying motor impairments and recovery potential. The limited number of participants within each diagnostic category prevented meaningful subgroup analyses, restricting condition-specific interpretation. Accordingly, the reported associations should be viewed as preliminary and applicable to mixed neurological populations rather than to any single pathology. Future studies with larger, diagnosis-stratified cohorts, particularly focused on high-prevalence groups such as stroke, are needed to validate and refine these observations. The heterogeneous composition of the neurological cohort represents an important methodological consideration. Although the included conditions share common gait-related impairments such as reduced postural control and restricted stride amplitude, their pathophysiological origins differ. Subgroup analyses were not statistically feasible given the limited number of participants within each diagnostic category; however, descriptive observations suggested that the overall pattern of associations between trunk stability and gait parameters was consistent within the group. Even so, this heterogeneity limits condition-specific interpretation, and future studies with larger, diagnosis-stratified samples are warranted to determine whether the observed relationships differ by neurological etiology. Additionally, the absence of a non-robotic control group limits causal inference regarding device-specific effects. Because robotic gait training constituted the standard of care in the participating clinical setting, allocating eligible patients to a non-robotic comparison arm was not feasible. As a result, the observed improvements should be interpreted as pre–post adaptations within the context of robotic rehabilitation rather than as evidence of treatment superiority relative to traditional therapy. This study was designed primarily to quantify segmental kinematic changes and examine the associations between trunk stability and spatiotemporal gait parameters, rather than to compare rehabilitation modalities. Nevertheless, future randomized or matched-control studies are needed to determine whether the magnitude and pattern of improvements differ meaningfully from those achieved with conventional gait therapy. Future research should employ larger, diagnosis-specific cohorts to better elucidate the underlying neuromuscular adaptations. Longitudinal follow-up studies could also determine the persistence of these kinematic improvements and their translation into functional mobility gains. Additionally, future work will focus on evaluating the long-term persistence of the gait and trunk stability improvements observed in this study. A longitudinal follow-up protocol is planned in which participants will undergo repeated gait assessments at 1-month, 3-month, and 6-month intervals after completing the robotic training program. These assessments will include the same kinematic waveform analyses and spatiotemporal measurements used in the present study, enabling direct comparison with immediate post-training outcomes. In addition, wearable sensor monitoring during daily walking will be incorporated to capture real-world gait behavior, providing ecological insight into whether neuromechanical gains translate into sustained functional mobility. Together, these longitudinal approaches will allow for a more comprehensive evaluation of the durability and clinical relevance of robotic gait rehabilitation outcomes.

Although several methodological limitations have been acknowledged, their implications for interpretation warrant further clarification. The small sample size reduces statistical power and increases the likelihood that observed effects reflect sample-specific rather than population-level trends. Diagnostic heterogeneity further complicates interpretation, as different neurological conditions exhibit distinct gait impairments and recovery profiles. Consequently, the reported associations between trunk stability and spatiotemporal improvements should be regarded as preliminary and descriptive, rather than generalizable across all neurological populations. The absence of a conventional rehabilitation control group also limits the ability to attribute changes specifically to robotic training, restricting conclusions to within-subject pre–post adaptations. While descriptive subgroup summaries have been added to enhance transparency, more definitive condition-specific patterns cannot be inferred from the present dataset. Future research employing larger, diagnosis-stratified cohorts and controlled study designs is required to validate the observed neuromechanical relationships and determine their consistency across clinical populations.

## 5. Conclusions

Clinically, the outcomes of this study underscore the role of robotic gait training as a complementary therapy in neurological rehabilitation. Enhancing trunk and hip control directly contributes to greater walking stability, improved stride confidence, and better transfer performance in daily life. The reduction in trunk sway is particularly encouraging, as it is closely associated with lower fall risk and increased independence in ambulatory tasks. Recent evidence highlights that trunk control is governed by a coordinated interaction of biomechanical stability and neural regulation, both of which are fundamental to efficient gait. Neural studies demonstrate that balance during walking emerges from dynamic sensorimotor integration, enabling the trunk to respond adaptively to perturbations and changing locomotor demands [28]. Complementary simulation work shows that maintaining upright trunk posture relies on robust neuromuscular coordination, with impaired axial muscle activation markedly reducing postural stability [29]. Biomechanical analyses further emphasize that trunk control is central to transitional and weight-shift tasks, as altered neuromuscular timing during movements such as sit-to-stand contributes directly to balance deficits, particularly in aging and neurologically impaired populations [30]. In gait, compensatory lateral trunk lean has been shown to redistribute joint loading and reflect underlying deficits in trunk stability, reinforcing the trunk’s critical role in maintaining efficient whole-body mechanics [31]. Related work examining postural control across task demands indicates that reduced trunk stability during walking is closely associated with diminished performance in unstable sitting conditions, highlighting the shared neural and biomechanical mechanisms that support axial control [32]. These insights collectively support the interpretation that the enhanced trunk motion and reduced lateral sway observed in our study likely reflect meaningful improvements in central trunk control mechanisms facilitated by robotic gait training.

The findings also suggest that sensor-based robotic systems such as the Tecnobody Smart Gravity Walker can serve not only as therapeutic tools but also as quantitative assessment platforms, enabling objective monitoring of recovery trajectories. The integration of motion capture and biofeedback capabilities provides clinicians with actionable metrics for individualized training optimization.

## Figures and Tables

**Table 1:** Summary of Paired Comparisons Between Pre- and Post-Rehabilitation Waveforms Across Kinematic Parameters.

Parameter	Mean ± SD (Pre)	Mean ± SD (Post)	Δ (Post–Pre)	t	*p*	d_x_ (Cohen’s d)	Interpretation
R Knee Flex–Ext (°)	20.33 ± 1.92	19.62 ± 1.87	−0.71	−1.56	0.123	−0.16	ns
L Knee Flex–Ext (°)	18.10 ± 1.88	18.02 ± 1.90	−0.07	−0.20	0.844	−0.02	ns
R Hip Flex–Ext (°)	6.81 ± 1.87	8.74 ± 1.90	+1.93	3.44	0.00084	0.34	↑ Significant increase
L Hip Flex–Ext (°)	5.77 ± 1.90	5.10 ± 1.85	−0.67	−1.87	0.064	−0.19	ns—trend toward decrease
Trunk Flex–Ext (°)	5.77 ± 1.89	8.82 ± 1.94	+3.04	9.49	1.4 × 10^−15^	0.95	↑ Highly significant increase
Trunk Lateral Flex (°)	0.32 ± 1.86	−0.32 ± 1.91	−0.63	−8.64	9.9 × 10^−14^	−0.86	↓ Highly significant decrease
Disp. COG (cm)	0.50 ± 1.88	1.35 ± 1.90	+0.85	15.15	1.6 × 10^−27^	1.51	↑ Highly significant increase

Data are expressed as mean ± standard deviation (SD). Paired-samples *t*-tests were conducted for each waveform parameter across 100 normalized stride points. Significance was set at *p* < 0.05. d_x_ denotes Cohen’s effect size for paired samples (0.2 = small, 0.5 = medium, 0.8 = large). Interpretation: R: Right; L: Left; ns = not significant; ↑ = significant post-rehabilitation increase; ↓ = significant post-rehabilitation decrease.

**Table 2:** Pre- and Post-Rehabilitation Comparison of Spatiotemporal and Kinematic Parameters Following 16 Sessions of Robotic Gait Training.

Parameter	Pre μ ± σ	Post μ ± σ	μ Diff	% Change	*p*	q (FDR)	Effect Size
Cadence (cycles/sec)	2.62 ± 7.6	0.43 ± 0.15	−2.177	11.7	1.0	1.0	0.5
Contact Time Left (s)	2.62 ± 1.44	2.49 ± 1.48	−0.129	9.1	0.786	0.903	−0.08
Contact Time Right (s)	2.59 ± 1.32	2.05 ± 0.99	−0.546	−5.1	0.247	0.557	−0.353
Load Symmetry (%)	5.81 ± 5.16	5.52 ± 4.57	−0.292	inf	0.837	0.903	−0.061
Step Length Left (cm)	17.08 ± 13.96	22.92 ± 16.18	5.833	67.4	0.022 *	0.119	0.769
Step Length Right (cm)	18.5 ± 15.62	27.83 ± 15.58	9.333	178.7	0.006 *	0.038	0.988
Vertical Displacement COG (cm)	1.64 ± 0.94	1.93 ± 0.85	0.292	33.8	0.323	0.672	0.298
L Hip flex-ext—ROM (°)	26.17 ± 8.46	28.82 ± 8.58	2.65	14.1	0.179	0.557	0.415
L Knee flex-ext—ROM (°)	35.95 ± 10.92	38.47 ± 14.78	2.517	9.7	0.514	0.902	0.195
R Hip flex-ext—ROM (°)	27.26 ± 9.76	25.91 ± 8.41	−1.35	−0.1	0.568	0.902	−0.17
R Knee flex-ext—ROM (°)	36.18 ± 16.18	36.91 ± 18.86	0.725	0.7	0.731	0.903	0.102
Trunk flex-ext—Average (°)	14.45 ± 7.68	9.5 ± 5.79	−4.95	−35.5	0.002 *	0.033	−1.129
ROM max (°)	17.93 ± 7.35	13.44 ± 6.28	−4.483	−24.8	0.005 *	0.038	−0.997
ROM min (°)	11.32 ± 7.49	5.78 ± 6.16	−5.533	−67.7	0.001 *	0.021	−1.323
Trunk lat- flex—Average (°)	−0.12 ± 3.49	−0.51 ± 2.88	−0.383	46.9	0.551	0.902	−0.178
ROM max (°)	3.23 ± 5.12	1.94 ± 3.15	−1.292	−73.4	0.215	0.557	−0.38
ROM min (°)	−2.57 ± 3.38	−3.1 ± 3.03	−0.533	−18.5	0.434	0.838	−0.234

* Statistically significant difference between pre- and post-rehabilitation assessments (*p* < 0.05).

## Data Availability

The datasets generated and analyzed during the current study are available in the following repository and can be accessed without restriction: https://1drv.ms/f/c/dee17576fbdaacfe/IgCTZtdFlCGdRoTrt0Z8XnVWAWYyYaYsXTmQZj-u0ulYGBc?e=Cl2GkS (accessed on 10 December 2025).

## Abbreviations

The following abbreviations are used in this manuscript:

RAGT	Robot Assisted Gait therapy
COG	Center of Gravity
ROM	Range of Motion
FDR	False Discovery Rate
